# Phenoxypropylamines: Synthesis and Antiulcer Evaluation

**DOI:** 10.3390/molecules14051818

**Published:** 2009-05-13

**Authors:** Hui Zhang, Bao-Yan Zhang, Qian-Yun Zhang, Dong-Mei Zhao, Jia-Mei Wang

**Affiliations:** 1 College of Sciences, Northeast University, Shenyang 110004, China; E-mail: baoyanzhang2005@163.com (B-Y.Z.); 2 Department of Chemistry, Shenyang Medical College, Shenyang 110034, China;; 3 School of Public Health, China Medical University, Shenyang 110001, China; E-mail: iamxiaoqian@126.com (Q-Y.Z.); 4 School of Pharmaceutical Engineering, Shenyang Pharmaceutical University, Shenyang 110016, China; E-mail: dongmeiz-67@163.com (D-M.Z.); 5 School of Medicinal Application & Technology, Shenyang Medical College, Shenyang 110034, China; E-mail: jinxing19890104@126.com (J-M.W.)

**Keywords:** roxatidine, synthesis, antiulcer agents, phenoxypropylamine, biological activity

## Abstract

We have synthesized a number of phenoxypropylamines from *N*-{3-[3-(1-piperidinylmethyl)phenoxy]propyl}chloroacetamide (**3**). All the products have been characterized by elemental analysis, ^1^H-NMR and MS. The biological activity effects of the title compounds were examined. From the biological activity results, we found that two of them showed significant gastric acid antisecretory activity.

## Introduction

The introduction of roxatidine acetate (**4**) as a H_2_-receptor antagonist for the control of peptic ulcer disease has been responsible for intense synthetic efforts by medicinal chemists in this therapeutic area to prepare highly efficacious drugs with greater potency and lower toxicity [1].

As part of our effort to explore a new antiulcer agent, this work was initiated with the goal of preparing a new compound which might possess potent gastric acid antisecretory and gastrointestinal protective activities with lower toxicity. In this paper we describe the preparation and antiulcer activity of compounds **6a-6f**. Roxatidine acetate (**4**) was synthesized by a series of reaction including Leukart reductive amination, acylation and substitution [2]. On the basis of the bioisosterism principle phenoxypropylamines were prepared from compound **3 **using primary amines [3]. The process is simple and moderate. The desired products were synthesized and identified on the basis of elemental analysis, ^1^H-NMR and MS. The title compounds **6a-6f** were also been evaluated for gastric acid antisecretory activity and the structure activity relationships of gastric acid antisecretory activity are also discussed.

## Results and Discussion

**Scheme 1:**
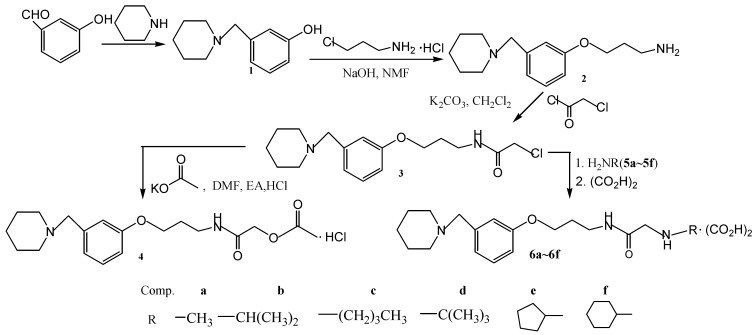
Synthesis of the title compounds **6a-6f**.

*N*-{3-[3-(1-Piperidinymethyl)phenoxy]propyl}chloroacetamide (**3**) was synthesized by a three-step reaction from *m*-hydroxybenzaldehyde. The hydrochloric acid salt of roxatidine acetic ester 4 was prepared in 28.8% yield by the reaction of **3** with AcOK [2]. Six phenoxypropylamine derivatives were then synthesized by the reaction of **3** with primary amines, followed by oxalic acid to obtain their corresponding salts **6**. The synthesis route is outlined in Scheme 1. 

### Biological evaluation [4,5]

**Figure 1:**
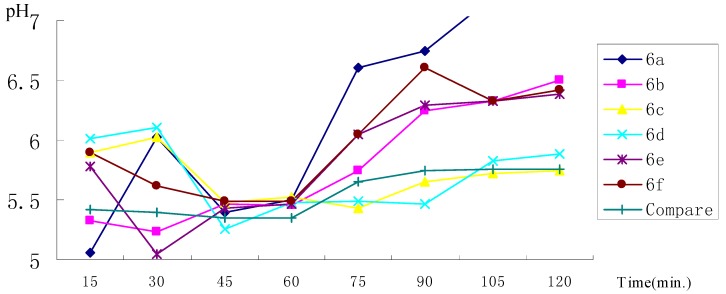
Phenoxypropylamine Effect on Gastric Acid Secretion in the Isolated Guinea Pig.

The effects of the title compounds **6a-6f** on gastric acid secretion are shown in Figure 1. The percentages of inhibition of gastric juice, GJ, 98.1%, 91.1%, 47.5%, 60.2%, 88.3% and 61.2%, respectively. As seen in the Figure, compounds **6a** and **6f** exhibited significant gastric acid antisecretory activity. That this gastric acid antisecretion efficacy was based on H_2_-receptor antagonist properties was confirmed by using the isolated guinea pig right atrial assay [7].

The calculation methods used In Figure 1 were as follows:

Acid output, AO = [H^+^]×0.05/ (л×1.25^2^×0.25) mol•cm^-2^•h^-1^
 = 10^-pH^×4×10^-2^ mol•cm^-2^•h^-1^
 = 10^-pH^×4×10^4^μmol•cm^-2^•h^-1^
       GJ = (Basic acid output, BAO – AO )/BAO × 100%

With regards to possible SAR, our preliminary assumption was to synthesize the compounds **6a** and **6b** by adding primary amine straight chains in order to enhance the ability of the molecules to inhibit gastric acid. We synthesized compounds **6c** and **6d** by adding a branched chain primary amine in order to enhance the flexibility of the molecules. The compounds **6e** and **6f** are the H_2_-receptor antagonists. 

## Conclusions

In summary, we have developed the method for preparing phenoxypropylamine derivatives of roxatidine acetate from *N*-{3-[3-(1-piperidinylmethyl)phenoxy]propyl}chloroacetamide. The protocol offers several advantages such as mild reaction conditions, short reaction times, easy isolation and good yields. Also two of them showed some potential effects on the inhibition of gastric juice.

## Experimental

### General

Melting points for the compounds were determined on a hot-stage microscope and are uncorrected. IR spectra were determined on a Nicolet 670FT-IR instrument in the range 4000─400 cm^-1 ^using the Smart OMNI-Sampler. ^1^H-NMR spectra were recorded in DMSO-d_6_ solution on a Bruker ARX-300 spectrometer operating at 300 MHz with TMS as the internal reference. Coupling constants (*J*) are expressed in Hz. MS spectra were obtianed using a Finnigan SSQ-710 spectrometer. The contents of carbon, hydrogen and nitrogen were determined on a Flash-1112 series elemental analyzer. Column chromatography was performed on silica gel (200-300 mesh) obtained from Qingdao Ocean Chemicals. Unless otherwise noted, all the materials were obtained from commercial sources and used without further purification.

*3-(1-Piperidinylmethyl)phenol* (**1**): 3-Hydroxybenzaldehyde (30.6 g, 0.25 mol) and 88 % formic acid (30.0 mL, 0.7 mol) were successively added to piperidine (50 mL, 0.5 mol) in a cool bath while the temperature was kept below 60°C. The mixture was then heated at 100°C for two hours and after cooling to 15°C it was diluted with water (75 mL). One gram of activated carbon was added and stirred for 1 h at room temperature for decolourizing. The carbon was filtered off, and the solution is made alkaline to pH = 9.0 by adding a 12% ammonium hydroxide solution. The product **1**, which crystallizes on standing at 15°C, was filtered and washed with water. When dried well the isolated compound **1 **weighed 36.02 g (79.3 %); m.p. 136-138°C. IR (KBr): 2900, 3400 cm^-1^; MS *m/z* (%): 191 (M^+^, 18), 107 (49), 84 (100); ^1^ H-NMR: 1.41-1.42 (d, 2H, *J*= 5), 1.56-1.59 (m, 4H, *J*=7), 2.43 (s, 4H), 3.41 (s, 2H), 6.44 (bs, 1H), 6.66-6.70 (d,2H, *J*= 8 ), 6.75-6.77 (d,1H, *J*= 8), 7.08-7.13 (t,1H, *J*=8) [2].

*N-{3-[3-(1-Piperidinylmethyl)phenoxy]propyl}amine* (**2**): 3-(1-Piperidinylmethyl)phenol (**1**, 9.6 g, 0.05 mol) and 3-chloropropylamine hydrochloride (8.40 g, 0.064 mol) were successively added to DMF (160 mL), then sodium hydroxide (28.4 g, 0.71 mol) was added. The reaction mixture is stirred at 85-90°C for two hours and filtered after cooling to 15°C. Potassium acetate (0.96 g, 0.01 mol) was added, and about 20 mL of DMF were distilled off under vacuum. The residue is diluted with water (40 mL) acidified with acetic acid to pH=4.6 and extracted with dichloromethane (50 mL). The aqueous layer is alkalinized to pH=9.5 with 12% ammonium hydroxide solution, extracted twice with dichloromethane (50 mL), dried with sodium sulfate, and filtered. This solution contains 12.04 g (97%) of **2** and is directly used in the next step without isolation. Oily substance; IR (film): 2925, 3300 cm^-1^; MS *m/z* (%): 248 (M+, 7.46), 190 (2.81), 165 (10.49), 107 (8.84), 98 (24.76), 84 (100); ^1^H-NMR δ: 1.36 (bs, 2H, *J*=5), 1.41-1.43 (d, 2H, *J*=5), 1.53-1.60 (m, 4H, *J*=5), 1.87-1.96 (m, 2H, *J*=7), 2.36 (s, 4 H ), 2.89-2.92 (t, 2H, *J*=7), 3.43 (s, 2H), 4.02-4.06 (t, 2H, *J*=7), 6.75-6.79 (dd, 1H, *J*=8), 6.87-6.89 (d, 2H, *J*=8), 7.17-7.22 (t, 1H, *J*=8) [2].

*N-{3-[3-(1-Piperidinylmethyl)phenoxy]propyl}chloroacetamide* (**3**): Dry potassium carbonate (11.3 g, 0.082 mol) was added to dried dichloromethane (100 mL) containing compound **2** (10.10 g, 0.04 mol). The mixture is cooled to 0-5°C, and chloroacetyl chloride (5.99 g, 0.053 mol) in dichloromethane (10 mL) were added. Thirty min later cold water (10 mL) was added, the organic layer washed twice with brine, dried, and filtered. The solvent is evaporated and the residue dried *in vacuo* to give 13.04 g (98.0%) of **3** as a colorless, partially crystallized oily substance. IR (film): 1670, 2940, 3000, 3400 cm^-1^; MS *m/z* (%): 324 (M+, 10), 240 (1.74), 190 (5.25), 134 (74.6), 84 (100); ^1^ H-NMR: 1.41-1.43 (d, 2H, *J*=5), 1.53-1.60 (m, 4H, *J*=5), 1.90 (s, 1H), 2.01-2.09 (m, 2H, *J*=7), 2.37 (s, 4 H), 3.44 (s, 2H), 3.51-3.57 (m, 2H, *J*=7), 4.06 (s, 2H), 4.07-4.11 (t, 2H, *J*=7), 6.77-6.80 (dd, 1H, *J*=8), 6.90-6.92 (d, 2H, *J*=8), 7.19-7.24 (t, 1H, *J*= 8) [2].

*N-{3-[3-(1-Piperidinylmethyl)phenoxy]propyl}acetoxyacetamide hydrochloride* (**4**): Chloroacetamide **3 **(1.07g, 0.0033 mol) is dissolved in DMF (40 mL), then dry potassium acetate (1.97 g, 0.0099 mol) was added. The mixture was stirred at 90-100°C for one hour, and most of DMF is distilled off under vacuum. The residue is evacuated at 40-50°C. Water (50 mL) acidified with acetic acid to pH=4.6 was added, and the solution is extracted with a portion of dichloromethane (20 mL). The acidic solution is decolourized with 1 g of activated carbon, alkalinized to pH=9.5 with a 12% ammonium hydroxide solution and then extracted twice with ethyl acetate (15 mL), washed with brine, and dried over sodium sulfate. The organic solution is treated with ethyl acetate saturated with hydrogen chloride at 0-5°C. The roxatidine acetate hydrochloride **4** separates as a white precipitate on cooling that agglomerates on standing. It is filtered and washed with dry ethyl acetate. After drying *in vacuo* 2.37 g (6.8 mmol) of **4** of 85.13% purity are obtained. Roxatidine acetate hydrochloride (**4): **m.p. 148-150°C; IR (CHCl_3_): 1675, 1755, 3000, 3430 cm^-1^; ^1^ H-NMR: 1.43-1.44 (d, 2H, *J*=5), 1.53-1.58 (m, 4H, *J*=5), 2.00-2.08 (m, 2H, *J*=7), 2.14 (s, 3H), 2.36 (s, 4H), 3.43 (s, 2H), 3.52-3.58 (q, 2H, *J*=7), 4.06-4.09 (t, 2H, *J*=7), 4.56 (s, 2H), 6.65 (bs, 1H), 6.77-6.80 (dd, 1H, *J*=8), 6.91-6.92 (d, 2H, *J*=8), 7.19-7.24 (t, 1H, *J*=8); Elemental anal. calcd. (%): C, 59.37; H, 7.55; N, 7.29. Found (%): C, 59.19; H, 7.86; N, 7.44; MS *m/z* (%): 348 (M^+^, 12.37), 305 (2.57), 222 (5.04), 190 (9.40), 158 (62.63), 84 (100) [2].

### Preparation and analytical and spectral data for compounds **6a** -**6f**

*N*-{3-[3-(1-Piperidinylmethyl)phenoxy]propyl}chloroacetamide (**3**, 0.0043 mol) are dissolved in acetonitrile (30 mL), then the appropriate primary amine (0.0086 mol) and dry potassium iodide (0.006 g) are added, and the mixture is stirred at 90-100°C for four hours. Most of acetonitrile is then distilled off under vacuum. Water (20 mL) acidified with acetic acid to pH=3.8 is added, and the solution is extracted with a portion of dichloromethane (20 mL). The acidic solution is decolourized with 1 g of activated carbon and alkalinized to pH=10.0 with a 12% ammonium hydroxide solution, then it is extracted twice with ethyl acetate (15 mL), washed with brine and dried over sodium sulfate. The organic solution is treated with ethyl acetate saturated with oxalate at 0-5°C. The phenoxypropylamine derivatives oxalate **6a-6f** are obtain as white precipitates [3].

*N-{3-[3-(1-Piperidinylmethyl)phenoxy]propyl}methylaminoacetamide* *oxalate* (**6a**): yield 62.8%; m.p. 132-134°C; ^1^H-NMR δ**:** 1.41-1.43 (d, 2H, *J*=5), 1.53-1.60 (m, 4H, *J*=5), 1.98 (t, 2H), 2.01-2.09 (m, 2H, *J*=7), 2.37 (s, 4 H), 2.54 (s,1H), 3.44 (s, 4H), 3.51-3.57 (m, 2H, *J*=7), 4.06 (s, 2H), 4.07-4.11 (t, 2H, *J*=7), 6.77-6.80 (dd, 1H, *J*=8), 6.90-6.92 (d, 2H, *J*=8), 7.19-7.24 (t, 1H, *J*=8); Elemental anal. calcd. (%) for C_18_O_2_N_3_H_29_: C, 67.68; H, 9.15; N, 13.15. Found (%): C, 67.48; H,9.20; N, 11.40. MS m/z (ESI): calcd. for C_18_O_2_N_3_H_29_ [M+H] 320.45, found 320.30.

*N-{3-[3-(1-piperidinylmethyl)phenoxy]propyl}isopropylaminoacetamide* *oxalate* (**6b**): yield 47.8%; m.p. 169-171°C; ^1^H-NMR δ: 0.85 (t, 3H), 1.26 (m, 2H), 1.42-1.44 (d, 2H, *J*=5), 1.52-1.61 (m, 4H, *J*=5), 1.93 (s, 1H), 2.01-2.09 (m, 2H, *J*=7), 2.39 (s, 4H), 2.87 (m, 4H), 3.45 (s, 2H), 3.52-.358 (m, 2H, *J*=7), 4.08 (s,2H), 4.09-4.13 (t, 2H, *J*=7), 4.10 (s, 1H), 6.97-6.99 (dd, 1H, *J*=8), 7.02-7.12 (d, 2H, *J*=8), 7.20-7.26 (t, 1H, *J*=8); Elemental anal. calcd. (%) for C_21_O_2_N_3_H_35_: C, 69.77; H, 9.76; N, 11.62. Found (%): C, 69.52; H,9.80; N, 11.60. MS m/z (ESI): calcd. for C_21_O_2_N_3_H_35_ [M+H] 362.54, found 362.30.

*N-{3-[3-(1-Piperidinylmethyl)phenoxy]propyl}n-butylaminoacetamide* *oxalate* (**6c**): yield 47.8%; m.p. 158-160°C. ^1^H-NMR δ: 0.95 (t, 6H), 1.26 (m, 2H), 1.42-1.44 (d, 2H, *J*=5), 1.52-1.61 (m, 2H, *J*=5), 1.93 (s, 1H), 2.01-2.09 (m, 2H, *J*=7), 2.39 (s, 2H), 2.87 (m, 3H), 3.45 (s, 2H), 3.52-.3.58 (m, 2H, *J*=7), 4.08 (s, 2H), 4.09-4.13 (t, 2H, *J*=7), 4.10 (s, 1H), 6.97-6.99 (dd,1H, *J*=8), 7.02-7.12 (d, 2H, *J*=8), 7.20-7.26 (t, 1H, *J*=8); Elemental anal. calcd. (%) for C_21_O_2_N_3_H_35_: C, 66.44; H, 9.20; N, 11.62. Found (%): C, 69.80; H,9.82; N, 11.59. MS m/z (ESI): calcd. for C_20_O_2_N_3_H_33_ [M+H] 348.50, found 348.30.

*N-{3-[3-(1-Piperidinylmethyl)phenoxy]propyl}tertiary-butylaminoacetamide* *oxalate* (**6d**): yield 76.4%; m.p. 129-131°C (from ethanol); ^1^H-NMR: 1.26 (m, 9H), 1.46-1.48 (s, 2H, *J*=5), 1.52-1.64 (s, 4H, *J*=5), 1.99 (s, 2H), 2.96 (s, 4H), 3.45 (s, 2H), 3.52-3.58 (m, 2H, *J*=7), 4.09 (s, 3H)**, **4.10-4.14 (t, 2H, *J*=7), 6.93-6.98 (dd, 1H, *J*=8), 7.04-7.30 (d, 2H, *J*=8), 7.18-7.25 (t, 1H, *J*=8), 8.8 (s, 1H); Elemental anal. calcd. (%) for C_21_O_2_N_3_H_35_: C, 69.77; H, 9.76; N, 11.62. Found (%): C, 69.62; H, 9.80; N, 11.39; MS m/z (ESI): calcd. for C_21_O_2_N_3_H_35_ [M+H] 362.54, found 362.40.

*N-{3-[3-(1-Piperidinylmethyl)phenoxy]propyl}cyclopentyaminoacetamide* *oxalate* (**6e**): yield 48.3%; m.p. 162-164°C (from ethanol); ^1^H-NMR: 1.42-1.43 (d, 2H, *J*=5), 1.48 (m, 4H), 1.56 (m, 4H, *J*=5), 1.91 (s, 3H), 2.01-2.09 (m, 4H, *J*=7), 2.38 (s, 4 H), 3.44 (s, 4H), 3.57 (m, 2H, *J*=7), 4.06 (s, 2H), 4.07-4.11 (t, 2H, *J*=7), 6.77-6.80 (dd, 1H, *J*=8), 6.90-6.92 (d, 2H, *J*=8), 7.19-7.24 (t, 1H, *J*=8); Elemental anal. calcd. (%) for C_22_O_2_N_3_H_35_: C, 70.74; H, 9.44; N, 11.25. Found (%): C, 70.54; H, 9.81; N, 11.32; MS m/z (ESI): calcd. for C_22_O_2_N_3_H_35_ [M+H] 374.55, found 374.00.

*N-{3-[3-(1-Piperidinylmethyl)phenoxy]propyl}cyclohexylaminoacetamide* *oxalate* (**6f**): yield 51.1%; m.p. 160-162°C(from ethanol); ^1^H-NMR: 1.42-1.43 (d, 2H, *J*=5), 1.48 (m, 4H), 1.56 (m, 4H, *J*=5), 1.91 (s, 3H), 2.01-2.09 (m, 4H, *J*=7), 2.38 (s, 4 H), 3.44 (m, 6H), 3.57 (m, 2H, *J*=7), 4.06 (s, 2H), 4.07-4.11 (t, 2H, *J*=7), 6.77-6.80 (dd, 1H, *J*=8), 6.90-6.92 (d, 2H, *J*=8), 7.19-7.24 (t, 1H, *J*=8); Elemental anal. calcd. (%) for C_23_O_2_N_3_H_37_: C,71.28; H, 9.62; N, 10.84. Found (%): C, 71.18; H, 9.32; N, 10.69. MS m/z (ESI): calcd. for C_23_O_2_N_3_H_37_ [M+H] 388.56, found 388.00.
